# Linking physiology to seascape: spatial patterns of energy intake in a highly mobile pelagic predator inferred from electronic tags

**DOI:** 10.1093/conphys/coag059

**Published:** 2026-08-12

**Authors:** Takaaki Hasegawa, Joe Scutt Phillips, Daniel W Fuller, Takaaki K Abe, Kei Okamoto, Yoshinori Aoki, Kazunori Kumon, Takeshi Eba, Takashi Kitagawa

**Affiliations:** Fisheries Resources Institute, Japan Fisheries Research and Education Agency, Fukuura, Kanazawa, Yokohama, Japan; Department of Living Marine Resources, Atmosphere and Ocean Research Institute, The University of Tokyo, Kashiwanoha, Kashiwa, Japan; Oceanic Fisheries Programme, Pacific Community, Promenade Roger Laroque, Nouméa, New Caledonia; Life History and Behavior Group, Inter-American Tropical Tuna Commission, La Jolla Shores Dr, La Jolla, CA, United States; College of Bioresource Science, Nihon University, Kamenoi, Fujisawa, Japan; Fisheries Resources Institute, Japan Fisheries Research and Education Agency, Fukuura, Kanazawa, Yokohama, Japan; Fisheries Resources Institute, Japan Fisheries Research and Education Agency, Fukuura, Kanazawa, Yokohama, Japan; Amami Field Station, Fisheries Technology Institute, Japan Fisheries Research and Education Agency, Sakiyamahara, Setouchi, Oshima, Kagoshima, Japan; Amami Field Station, Fisheries Technology Institute, Japan Fisheries Research and Education Agency, Sakiyamahara, Setouchi, Oshima, Kagoshima, Japan; Graduate School of Frontier Sciences, The University of Tokyo, Kashiwanoha, Kashiwa, Japan

**Keywords:** Archival tag, geostatistical model, heat increment of feeding, *Thunnus albacares*, yellowfin tuna

## Abstract

Spatial heterogeneity in energy acquisition of marine animals shapes regional variation in demographic processes and has important implications for conservation and management. However, quantifying energy intake in large pelagic predators and evaluating its broad-scale spatial structure remain extremely challenging. Here, we examined the spatial distribution of energy intake in yellowfin tuna (*Thunnus albacares*) across the tropical Pacific Ocean by coupling archival tagging data with a geostatistical model. We first conducted a feeding experiment to establish the relationship between the heat increment of feeding and ingested energy in yellowfin tuna (*N* = 20) using archival tags. This relationship was then applied to archival tagging data from wild fish (*N* = 36) to estimate their field energy intake. We then predicted spatial variation in foraging probability and positive energy intake with a geostatistical model and explored potential environmental drivers. Our results indicated a clear difference in energy intake, with higher values observed in the eastern Pacific Ocean (EPO) compared to the western Pacific Ocean (WPO). Thermocline depth had a significant negative effect on foraging probability (${\alpha}$ = −0.58, 95% CI: −1.08 to −0.07), indicating that feeding was more likely in regions with a shallower thermocline. Model-based predictions showed a consistent eastward increase in mean energy intake, with values in the EPO reaching ~2.2 times those in the WPO. These results suggest that strong east–west contrasts in oceanographic structure across the tropical Pacific Ocean systematically shape feeding opportunities and energy acquisition in yellowfin tuna. Our findings provide physiological insight into mechanisms driving spatial variation in key demographic processes such as growth, with important implications for conservation and management of this commercially important species in a changing ocean.

## Introduction

The amount of energy that animals acquire from the environment shapes their physiological state and constrains survival, growth, and reproduction (Kooijman, 2010). Therefore, variation in energy intake ultimately influences animal movement, distribution, and population growth rates (Pirotta, 2022). Ingested energy depends on several extrinsic (e.g. prey density and distribution, nutritional value and composition) and intrinsic factors (e.g. foraging effort and success at catching prey) and often shows spatial heterogeneity in marine environments (Arostegui *et al.,* 2023; Booth *et al.,* 2023). Such spatial heterogeneity in energy intake can generate regional differences in life-history characteristics, which in turn affect population dynamics of marine animals. Understanding the spatial distribution of energy intake is therefore essential for interpreting spatial variation in population growth rate and for informing effective conservation and management from a physiological perspective (Wilson *et al.,* 2012). However, direct measurement of energy intake in free-ranging large pelagic predators remains extremely challenging (Naito *et al.,* 2013), limiting our capacity to evaluate potential spatial heterogeneity in energy acquisition in the wild.

Recent advances in biologging technologies have enabled high-resolution recordings of environmental conditions and some physiological variables in free-ranging fishes (Kitagawa *et al.,* 2004; Hussey *et al.,* 2015; Watanabe and Papastamatiou, 2023). Electronic archival tags, in particular, have been widely used in tunas to study the interaction between their behaviour, and thermal physiology (Kitagawa *et al.,* 2001, 2006, 2007, 2022; Abe *et al.,* 2025; Aoki *et al.,* 2025). Regional endothermy allows many tuna species to retain metabolic heat and maintain the temperature of certain tissues (red muscle, visceral organs, brain, and eyes) above water temperature (Bernal *et al.,* 2017). This physiological trait allows a postprandial increase in visceral temperature, known as the heat increment of feeding (HIF), to be detected from temperature records from archival tags (Clark *et al.,* 2008). HIF arises from increased metabolic heat production associated with the digestion and assimilation of ingested food (Clark *et al.,* 2010). Laboratory studies have demonstrated a clear relationship between ingested energy and the magnitude of HIF in captive Pacific bluefin tuna [*Thunnus orientalis*, (Whitlock *et al.,* 2013)] and skipjack tuna [*Katsuwonus pelamis*, (Aoki *et al.,* 2017)]. Based on this evidence, field studies have used HIF profiles derived from archival tagging data as a proxy for energy intake in wild tunas (Whitlock *et al.,* 2015; Aoki *et al.,* 2017; Muhling *et al.,* 2022). By simultaneously recording environmental variables, behaviour and location of individuals, archival tags are powerful tools to explore the potential drivers shaping the spatial distribution of energy intake in natural environments (Whitlock *et al.,* 2015; Aoki *et al.,* 2017; Muhling *et al.,* 2022).

The tropical Pacific Ocean provides an ideal system to evaluate how environmental heterogeneity structures the energy intake of tropical tunas using electronic tagging data. This region is characterised by a clear longitudinal gradient in oceanographic conditions. The western tropical Pacific is dominated by a warm pool with a deep thermocline and oligotrophic conditions, whereas the eastern tropical Pacific is influenced by upwelling, resulting in a shallower thermocline and higher primary production (Chavez *et al.,* 1996; Irigoien *et al.,* 2014). These contrasting patterns in vertical thermal structure and ecosystem productivity can drive prey abundance and vertical distribution (Lehodey *et al.,* 2008, 2010), thereby influencing feeding opportunities for tropical tunas. In addition, this region has been the focus of extensive electronic tagging programmes for tropical tunas, and archival tagging data are available across the tropical Pacific (Leroy *et al.,* 2015; Schaefer and Fuller, 2022). Applying the HIF-based approach to these tagging data allows us to explore the link between longitudinal environmental gradients and spatial patterns of energy intake in tropical tunas.

Yellowfin tuna (*Thunnus albacares*) is one of the most ecologically and commercially important tuna species in the tropical Pacific Ocean. It primarily inhabits epipelagic waters and exhibits diel vertical movements, remaining near the surface at night and diving repeatedly during the day for foraging (Sund *et al.,* 1981; Brill, 1994). Electronic tagging studies have shown that daytime depth is closely associated with thermocline depth (Block *et al.,* 1997), suggesting that habitat depth is shallower in the eastern Pacific Ocean (EPO) and deeper in the western and central Pacific Ocean (WCPO). Such longitudinal differences in vertical habitat use can influence prey encounter and feeding conditions and may lead to spatial differences in energy intake across the tropical Pacific Ocean. Previous studies have also reported regional differences in growth patterns of yellowfin tuna between the WCPO (Farley *et al.,* 2020) and the EPO (Wild, 1986). Estimating the spatial distribution of energy intake can therefore provide a physiological link between environmental heterogeneity and regional variation in growth.

This study aimed to estimate field energy intake of wild yellowfin tuna using archival tags and predict its spatial distribution across the tropical Pacific Ocean. First, we conducted a feeding experiment with archival-tagged yellowfin tuna in captivity to establish the relationship between the magnitude of the HIF and energy intake. This relationship was then applied to archival tagging data collected in the WCPO and the EPO to estimate field energy intake of yellowfin tuna. Finally, we used geostatistical models to examine potential drivers of energy intake and to predict its spatial distribution across the tropical Pacific Ocean.

## Materials and methods

### Ethics statement

All applicable international, national, and institutional guidelines for the care and use of animals were followed. All procedures performed in studies involving animals were in accordance with the Guidelines for Proper Conduct of Animal Experiments (Science Council of Japan) and were approved by the Animal Experiment Committee of the Japan Fisheries Research and Education Agency (Permit No. FRI-R6-25).

### Feeding experiment

The experiment was conducted at the Amami Field Station located on Kakeroma Island in the Amami Archipelago. This facility is managed by the Fisheries Technology Institute of the Japan Fisheries Research and Education Agency. We used two round offshore cages (cage A for holding and cage B for the feeding experiment) with diameters of 10 m and nominal depths of ~10 m, varying with tidal conditions.

We collected juvenile yellowfin tuna (fork length: 35–50 cm) from the coastal waters of the Amami Archipelago (27°24′N–28°45′N, 128°24′E–129°56′E) on 26 January 2024 using a local pole-and-line fishing boat. The fish were caught using a rod and reel equipped with barbless hooks and immediately transferred to a rectangular tank on the boat without direct physical contact. Upon arrival at the facility, the fish were individually transferred from the tank to cage A using a rubber-mesh net. A total of 59 individuals were transported to cage A and acclimated for 2 weeks. During the acclimation period, the fish were fed daily with sand eels (*Ammodytidae*) and krill (*Euphausiidae*).

Prior to tagging, wild fish present in cage B (~ >10 cm in length) were removed as thoroughly as possible to minimize the possibility of unrecorded feeding events during the experiment. On 13 February 2024, 25 fish were surgically implanted with archival tags (LAT2810, Lotek Wireless Inc., Ontario, Canada) into the peritoneal cavity. These tags were programmed to measure ambient water temperature and peritoneal temperature every 1 s. The tagging procedure was as follows: a yellowfin tuna was recaptured from cage A using a rod and reel with barbless hooks and lifted from the water using a rubber landing net and then placed on a urethane foam mat. An incision ~1 cm long was made with a blade anterior to the anus. The tag was inserted deep into the peritoneal cavity to record the temperature proximal to the stomach. The incision was closed with two staples using a staple gun. All procedures were performed in <1 min. After tagging, fish were transferred to cage B and allowed to recover from potential effects of handling and tagging. During the recovery period, we monitored the fish daily for their feeding behaviour and general condition and confirmed that all fish resumed normal feeding behaviour prior to the experiment. This recovery period was 5 days in the present study.

Feeding experiments were conducted in cage B between 19 and 24 February 2024. Food items consisted of sand eels or krill, which were thawed to the water temperature of the sea cage prior to feeding. During the experiment, yellowfin tuna were fed daily food amounts ranging from 500 to 2500 g, corresponding to target daily rations (daily food consumption expressed as a percentage of body mass) of ~2–10% (Table S1). These target daily rations were selected to be comparable to field estimates for yellowfin tuna reported in previous studies (Varghese and Somvanshi, 2016). Food consumption was recorded at the cage level rather than for individual fish. Individual food consumption was therefore estimated by dividing the total food amount by the number of tagged fish present in cage B, following the method of Aoki *et al.* (2017). These values should be interpreted as per-capita estimates based on cage-level consumption and include uncertainty arising from among-individual variation in actual food intake. The consumed food was converted into meal energy (kcal) based on a calorie table (1.1 kcal g^−1^ for sand eel and 0.94 kcal g^−1^ for krill) from the food composition database (MEXT, 2023). Although the sea cage was located in open water and the presence of wild fish could not be entirely excluded, our tagged fish consistently fed on the supplied food. No apparent feeding behaviour towards prey other than the supplied diet was observed during the experiment. We therefore assumed that unrecorded feeding was unlikely to have substantially influenced the HIF responses. At the end of the experiment, tagged fish were recaptured by rod and reel, and their fork length and body weight were recorded (Table S2). The position of each archival tag within the peritoneal cavity was examined to confirm whether the tag was in contact with the stomach.

### Quantification of HIF

Quantification of HIF requires the estimation of a baseline body temperature representing the expected body temperature when there is no visceral warming due to feeding (Whitlock *et al.,* 2013). We followed the approach described by Aoki *et al.* (2017), who demonstrated its applicability to skipjack tuna. This method uses a heat budget model (Holland *et al.,* 1992) to estimate baseline body temperature while accounting for thermal responses to fluctuations in ambient water temperature. Because tropical tunas such as yellowfin and skipjack tuna lack the well-developed heat conservation mechanisms observed in bluefin tuna, this approach is particularly suitable for accurately estimating baseline body temperature in these species. Body temperature dynamics in the absence of feeding were assumed to follow a first-order heat balance equation:


(1) \begin{equation*} \frac{T_b\left(t+1\right)-{T}_b(t)}{\Delta t}=\mathrm{\lambda} \left[{T}_w(t)-{T}_b(t)\right]+\frac{d{T}_m}{dt}, \end{equation*}

where ${T}_b(t)$ and ${T}_w(t)$ represent observed body temperature and ambient water temperature at time $t$, respectively. The left-hand side describes the observed rate of change in body temperature per unit time, with $\Delta t$ denoting the observation interval. The parameter $\lambda$ represents the whole-body heat transfer coefficient (s^−1^) between the fish and the surrounding water, whereas $d{T}_m/ dt$ represents a constant rate of temperature change due to metabolic heat production (°C s^−1^) unrelated to feeding.

The model was fitted to data collected during a non-feeding period from 12:00 on 17 February to 12:00 on 19 February 2024 to estimate $\lambda$ and $d{T}_m/ dt$ for each individual. Using the estimated parameters, we predicted the baseline body temperature during the experiment. Following previous studies (Whitlock *et al.,* 2013; Muhling *et al.,* 2022), we applied a centred 1-h moving average to water temperature, observed body temperature, and estimated baseline body temperature to minimize the influence of high-frequency temperature fluctuations.

To quantify the increase in body temperature associated with feeding, we defined HIF as the difference between the observed body temperature and the estimated baseline body temperature. HIF was quantified as the time integral of this temperature difference following feeding. We calculated this metric, referred to as the HIF area ($\mathrm{\phi}$; °C·min) as follows:


(2) \begin{equation*} {\displaystyle \begin{array}{c}\mathrm{\phi} ={\int}_{t_s}^{t_e}\big({T}_b(t)-\hat{T_b}(t)\big)\; dt,\end{array}} \end{equation*}

where ${t}_s$ and ${t}_e$ denote the start and end of the feeding-induced thermal response, respectively. $\hat{T_b}(t)$ represents the estimated baseline body temperature. We calculated the HIF area by integrating only positive deviations, that is, periods when body temperature exceeded the baseline, using feeding time as the reference. Feeding was conducted once per day, and no additional feeding occurred before digestion was completed. We therefore calculated HIF areas independently for each feeding event.

### Statistical analysis for the feeding experiment

All statistical analyses were conducted using R [version 4.4.2; (R Core Team, 2025)]. To quantify the relationship between meal energy and HIF area, we developed generalized linear mixed-effects models (GLMMs) while accounting for individual variation. Because HIF area is a continuous variable that takes only positive values, we assumed a Gamma error distribution with a log link function. HIF area for observation $j$ from individual $i$ can be modelled as follows:


(3) \begin{equation*} {\displaystyle \begin{array}{c}{\phi}_{ij}\sim \mathrm{Gamma}\left({\mu}_{ij},k\right)\!,\end{array}} \end{equation*}

(4) \begin{equation*} {\displaystyle \begin{array}{c}\log \left(\mu \right)=\mathbf{X}\boldsymbol{\mathrm{\beta} } +\mathbf{Zu},\end{array}} \end{equation*}

where ${\mu}_{ij}$ is the vector of expected HIF area and $k$ is the dispersion parameter. $\mathbf{X}$ and $\boldsymbol{\mathrm{\beta}}$ denote the design matrix and coefficient vector for fixed effects, respectively. $\mathbf{Z}$ represents the design matrix for random effects, and $\mathbf{u}$ is the vector of random effects associated with each individual and is assumed to follow a normal distribution.

To evaluate the effects of meal energy on HIF area, we constructed a set of candidate models with different fixed- and random-effects structures (Table 1). Fixed effects included meal energy and food type (sand eel or krill). Random effects included individual-specific intercepts based on fish ID, as well as individual differences in sensitivity to meal energy through random slopes.

**Table 1:** Model selection results for GLMMs fitted to HIF area (°C·min day^−1^)

**Response variable**	**Model**	**Fixed effects**	**Random effects**	**Log likelihood**	**AIC**
HIF area	M1	energy	id	−465.7	944.1
	M2	energy + food	id	−467.8	942.1
	M3	energy × food	id	−465.7	944.4
	M4	energy + food	energy × id	−465.0	945.3

Candidate models differ in their fixed- and random-effects structures. Fixed effects include daily meal energy (energy, kcal day^−1^), food type (food; sand eel or krill), and their interaction. Random effects include individual-specific intercepts (id) and, where applicable, individual-specific random slopes for meal energy (energy × id). Model performance was evaluated using the AIC.

We estimated the model parameters using the R package *glmmTMB* (Brooks *et al.,* 2017), which allows flexible implementation of GLMMs with non-Gaussian error distributions together with complex random-effects structures. Prior to model fitting, meal energy was standardized to a mean of 0 and a SD of 1 to improve convergence of the optimization algorithm. We evaluated relative model performance based on Akaike’s Information Criterion [AIC, (Akaike, 1974)] and selected the model with the lowest AIC as the supported model that best explained the relationship between meal energy and HIF area.

### Tagging data from wild fish

Archival tagging data from wild yellowfin tuna were obtained from tagging programmes conducted by the Pacific Community (SPC) and the Inter-American Tropical Tuna Commission (IATTC) between 2006 and 2022. In total, data from 36 individuals were used in this study (Table S3). Locations at release and recapture covered a broad longitudinal range across the tropical Pacific Ocean (Fig. 2A), including the western Pacific Ocean (WPO; 2°S–9°S, 146°E–176°E), the central Pacific Ocean (CPO; 5°S–7°N, 179°W–177°E), and the EPO (16°N–3°S, 140°W–79°W). The SPC tagging programme was conducted in the WPO and CPO, where fish were captured primarily using pole-and-line or sportfishing gear. The IATTC tagging programme was conducted in the EPO, where fish were captured using trolling, handline, or pole-and-line methods. Detailed tagging and handling procedures are described elsewhere for the SPC tagging programme (Leroy *et al.,* 2015) and the IATTC tagging programme (Schaefer and Fuller, 2022). Both programmes used similar methods to surgically implant archival tags into the peritoneal cavity of each fish, following recommended best practices for the intracoelomic implantation of telemetry tags in tropical tunas (Leroy *et al.,* 2023). Tags recorded pressure (depth in m), ambient water temperature, peritoneal temperature, and relative ambient light intensity at sampling intervals of 10, 20, 30 or 60 s, depending on tag type and deployment. To facilitate detection and recovery of archival tags upon recapture, each fish was also fitted with a conventional plastic dart tag inserted at the base of the dorsal fin. Fork length was measured at both release and recapture.

Depending on data availability and tag performance, daily positions for each tagged fish were estimated using one of three approaches: *TrackIt* (Lam *et al.,* 2010), *ukfsst* (Lam *et al.,* 2008) or Wildlife Computers light-based geolocation (*WLC*). Both *TrackIt* and *ukfsst* are state-space modelling approaches that estimate the most probable movement paths of tagged fish by integrating light-based geolocation with sea surface temperature (SST) data. In both methods, movement is typically represented as a random walk, and uncertainty in light-based geolocation is explicitly accounted for within a statistical framework. *TrackIt* uses a particle-filter framework, whereas *ukfsst* incorporates SST within a unified state-space model using an unscented Kalman filter, allowing non-linearities and observation error to be handled internally. In contrast, *WLC* estimates daily positions using a maximum likelihood procedure based on light-level geolocation while incorporating SST, but it does not explicitly integrate a movement process within a state-space modelling framework, as implemented in *TrackIt* and *ukfsst*. Detailed procedures for each method are described in Schaefer and Fuller (2022) for *ukfsst* and Leroy *et al.* (2015) for *TrackIt*.

Each individual was assigned a single geolocation method for the entire deployment period. For individuals for which light-based geolocation could not be performed for the entire deployment due to abnormal or missing light records, daily positions were estimated by linear interpolation between the release and recapture locations. For individuals in which geolocation was successful only for part of the deployment period, missing positions after the last reliable estimate were interpolated linearly between the final estimated position and the recapture location. In cases of short gaps in otherwise continuous daily position estimates, missing locations were interpolated linearly using latitude and longitude estimates from adjacent days. To reduce the influence of high-frequency noise and residual geolocation error, a centred 7-day moving average was applied to the daily latitude and longitude time series (Braun *et al.,* 2018).

Prior to analysis, we removed data from 1 day after release to minimize potential effects of fish handling stress (Leroy *et al.,* 2023). For individual L02783, irregularities in external water temperature records limited reliable data to the first 246 days post-release, so we excluded data collected thereafter. Similarly, for individual 270134, irregularities in depth records restricted usable data to the first 255 days post-release, and we excluded data beyond this period. After quality control, the number of observation days per individual ranged from 16 to 353 (mean = 138). In total, we retained 4736 daily observations from 36 individuals for analysis. For each fish, we quantified the proportion of analysis days with available light-based geolocation estimates. Across all individuals, geolocation was available for 84.3% of analysis days (3993 of 4736 days). Most fish without geolocation data were short-duration release-to-recapture records (<50 days) in which release and recapture occurred within the same broad region (WPO, CPO, or EPO). One longer record without geolocation spanning ~1 year (fish 340 037; WPO) also showed very similar release and recapture positions. For this fish, the tag-derived thermal environment remained consistent with the WPO throughout the entire deployment period (Fig. S1), with little evidence of a sustained shift towards the thermal characteristics of other regions, supporting retention of this record as a WPO observation rather than a cyclic migration through another region (see more details in Supplementary Materials). Given that our analyses were conducted at the basin scale, these records were considered unlikely to bias the regional comparisons.

### Thermal structure analysis

To quantify the vertical thermal structure experienced by tagged yellowfin tuna, we analysed temperature and depth data to construct temperature–depth (T–D) profiles. For each individual, we first extracted observations on a daily basis and grouped depth records into 10 m depth bins from 0 to 700 m. Within each depth bin, we calculated the mean ambient temperature on that day and used these values to construct daily T–D profiles. This approach reduced the influence of uneven sampling frequency across depths while capturing the representative daily vertical structure of water temperature. Based on the daily T–D profiles, we calculated the vertical temperature gradient ($dT/ dz$) between adjacent depth bins. For each day, we identified the maximum vertical gradient and defined the thermocline as the depth range where the gradient exceeded 50% of this maximum value. We recorded the upper and lower bounds of this range as the upper and lower limits of the thermocline for that day. For each individual, we then calculated the median upper and lower thermocline depths across the analysis period and defined the representative thermocline depth for each fish as the mean of these two median values.

### Estimation of energy intake based on HIF

To estimate field energy intake of wild yellowfin tuna, we applied the relationship between ingested energy and HIF area obtained from the feeding experiment. Unlike captive fish, wild fish experience a wide range of ambient temperatures as they move through their environment, and archival tag records often span long time periods. Previous studies have shown that the whole-body heat exchange coefficient ($\mathrm{\lambda}$) can vary over time (Teo *et al.,* 2007). In contrast, the rate of metabolic heat production ($d{T}_m/ dt$) remains relatively stable during diving and surfacing behaviour in tunas (Holland *et al.,* 1992; Kitagawa *et al.,* 2006). We first classified the time series data for body temperature as warming and cooling phases by examining whether the differences between the data points (${T}_b\left(t+1\right)-{T}_b(t)$) were positive or negative. Consecutive warming or cooling phases were grouped into continuous segments and used for parameter estimation. For each focal day, we estimated $\lambda$ using a 7-day moving window that included the focal day and the three preceding and following days and used this parameter as representative of that day. Based on the estimated parameters, we predicted baseline body temperature and quantified daily HIF area following the same procedure as described in the feeding experiment. We then estimated daily energy intake by applying the relationship established in the feeding experiment to the HIF area. When the daily HIF area fell below the intercept of the relationship, we regarded the value as noise and set energy intake to zero, assuming that potential feeding events were below the detection threshold of this approach. Because HIF area is also known to vary with ambient water temperature (Whitlock *et al.,* 2013), and the tropical Pacific Ocean is characterised by the longitudinal variation in thermal conditions, uncorrected HIF-based estimates could be confounded by regional differences in ambient temperature. To address this, we derived a temperature-dependent correction factor (Fig. S2) from the temperature–HIF relationship reported for Pacific bluefin tuna by the previous study (Table S4) calculated as the ratio of the fitted slope at the calibration temperature (22°C) in our feeding experiment to the fitted slope at daily mean ambient temperature for each fish. This correction factor was applied to each estimate of daily energy intake, and the resulting temperature-corrected values were used in all subsequent analyses (see more details in Supplementary Materials).

### Geostatistical analysis of energy intake

To examine spatial variation in estimated energy intake, we calculated the median energy intake for each fish within each 1° × 1° spatial grid cell. Using the median values reduced the influence of high-frequency daily variability and outliers, thereby summarizing representative spatial patterns of habitat use and energy intake for each individual. We used tag-derived thermocline depth and satellite-based chlorophyll a concentration as environmental covariates. Chlorophyll a data were obtained from Aqua MODIS satellite observations provided by the NASA Ocean Biology Processing Group (Level 3, monthly composites, 9 km spatial resolution). For each observation day, we identified the corresponding year, month, and 1° × 1° grid cell based on the estimated daily location and assigned the monthly mean chlorophyll a concentration at the centre of that grid cell as the environmental condition for that day. Thermocline depth estimates were derived from archival tag data. This approach allowed temporal variation in environmental conditions to be incorporated through the time-specific covariates assigned to each observation day, while the use of within-cell median energy intake emphasised broad-scale spatial structure over short-term temporal variability. Accordingly, our geostatistical analysis was designed to characterise basin-scale spatial patterns rather than seasonal or inter-annual temporal dynamics.

To accurately characterise spatial variation in energy intake and evaluate its environmental drivers, several challenges associated with the structure of the data needed to be addressed. First, daily estimates of energy intake included zero values, reflecting days when yellowfin tuna may have not fed. As the data therefore consisted of a mixture of zero values and positive continuous values, they could not be adequately represented by a single continuous probability distribution. Second, the number of individuals and observations varied markedly among locations, leading to uneven sampling intensity across space that could bias energy intake estimates. Third, repeated observations were obtained from the same individuals over time, violating the assumption of independence among observations if individual effects were ignored. Finally, spatial autocorrelation was expected in energy intake due to shared environmental conditions among nearby locations, such that observations close in space were likely to be more similar than those farther apart. Failure to account for this spatial dependence could result in biased parameter estimates and underestimated uncertainty.

To address these issues simultaneously, we developed a spatial delta-lognormal GLMM using the R package *sdmTMB* (Anderson *et al.,* 2022). The model incorporates spatial random effects to account for spatial autocorrelation, as well as individual-level random effects to accommodate repeated measurements from the same fish. The delta approach models two processes separately: (i) the probability of observing non-zero energy intake (encounter process) and (ii) the magnitude of positive energy intake conditional on feeding (positive process). Energy intake for observation $i$ was modelled as follows:


(5) \begin{equation*} {\displaystyle \begin{array}{c}{E}_i=\left\{\begin{array}{@{}c}0\\{}\mathrm{lognormal}\left({\mu}_i,{\sigma}^2\right)\end{array}\right.\!\!\!\!,\end{array}} \end{equation*}

where zero observations occur with probability $1-{p}_i$ and positive observations follow a lognormal distribution with probability ${p}_i$. The encounter probability ${p}_i$ and the expected value of positive energy intake ${\mu}_i$ were described by the following linear predictors:


(6) \begin{equation*} {\displaystyle \begin{array}{c}\mathrm{logit}\left({p}_i\right)={\alpha}_0+{\alpha}_1{\mathbf{X}}_{\mathbf{i}}+{\omega}_1\left({s}_i\right)+{b}_{1,i},\end{array}} \end{equation*}

(7) \begin{equation*} {\displaystyle \begin{array}{c}\log \left({\mu}_i\right)={\beta}_0+{\beta}_1{\mathbf{X}}_{\mathbf{i}}+{\omega}_2\left({s}_i\right)+{b}_{2,i},\end{array}} \end{equation*}

where ${\alpha}_0$ and ${\beta}_0$ are the intercepts; ${\alpha}_1$ and ${\beta}_1$ are the regression coefficients. ${\mathbf{X}}_{\mathbf{i}}$ represents environmental covariates, ${\omega}_1\left({s}_i\right)$ and ${\omega}_2\left({s}_i\right)$ denote a spatial random effect associated with location ${s}_i$ and, ${b}_{1,i}$ and ${b}_{2,i}$ are individual-specific random effects and are assumed to follow a normal distribution. The spatial random effect was formulated as a continuous Gaussian random field with a Matérn covariance structure and estimated using a triangular mesh constructed over the study region (the number of nodes = 100). Mesh resolution was selected to capture spatial structure while avoiding excessive model complexity.

For all the candidate models, sanity checks on the items listed below were performed by the *sanity* function of *sdmTMB*; the non-linear minimizer suggests successful convergence, the Hessian matrix is positive definite, no extreme or very small Eigen values are detected, no gradients with respect to fixed effects are greater than 0.001, no fixed-effect SE are reported as NA, no fixed-effect SE look unreasonably large, no sigma parameters are <0.001, and the range parameter does not look unreasonably large. We evaluated model performance based on AIC and selected the model that best explained daily energy intake.

To predict the spatial distribution of energy intake, we generated predictions on 1° × 1° grids covering the study region while excluding individual random effects. This approach allowed prediction of spatial variation in encounter probability, positive energy intake, and integrated energy intake. We further summarized predicted values longitudinally and calculated 95% confidence intervals based on model simulations to evaluate east–west spatial trends across the tropical Pacific Ocean.

## Results

### Feeding experiments

**Figure 1:**
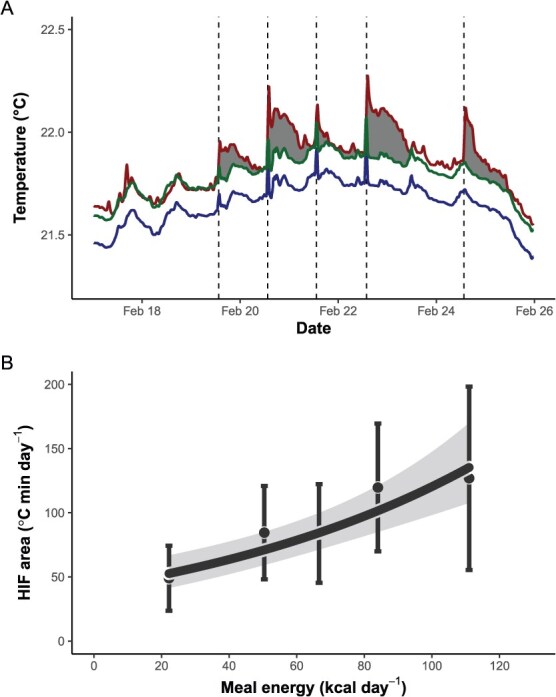
Results for feeding experiment. (**A**) Example time series of ambient temperature (blue), body temperature (red), and estimated baseline body temperature (green) from a tagged yellowfin tuna (ID: 2140213). Dashed lines indicate the start of each feeding trial. Grey shaded area represents the HIF, defined as the area where body temperature exceeds the baseline body temperature. (**B**) Relationship between HIF area (°C·min day^−1^) and meal energy (kcal day^−1^) across all feeding events (*N* = 40). The solid line indicates the fitted mixed-effects relationship, with the dark band showing the 95% confidence interval for the mean prediction

Of the 25 archival-tagged yellowfin tuna, 20 individuals kept in captivity (fork length: 37.6–45.7 cm, body weight: 0.9–1.8 kg, Table S2) provided continuous tag records throughout the feeding experiment and were included in the analysis. Data from the remaining five tagged fish were not used because the tag records were either not recoverable or had terminated before the experiment. During the experiment, yellowfin tuna experienced water temperature ranging from 21.4 to 22.0°C (mean ± SD: 21.7 ± 0.12°C). During non-feeding periods, body temperature remained on average 0.17 ± 0.06°C above ambient water temperature. The baseline body temperature estimated by the heat budget model was generally consistent with the observed body temperature, with a mean difference of 0.04 ± 0.06°C. After feeding, body temperature increased above the baseline level and reached a peak increase of 0.26 ± 0.10°C. The elevated body temperature persisted for 22.0 ± 2.1 h before declining towards the baseline level (Fig. 1A). The duration for which body temperature remained above the baseline varied with meal energy (Fig. 1B). In some cases, body temperature remained elevated until shortly before the subsequent feeding trial. We defined these periods of postprandial increase in body temperature as the HIF and evaluated the effect of meal energy on individual HIF area using GLMMs.

Model selection based on AIC indicated that the model including meal energy and food type as fixed effects and individual-specific intercepts as random effects (M2) was the most supported (Table 1). However, the difference in AIC between M2 and the model including only meal energy as a fixed effect (M1) was relatively small (ΔAIC = 1.92), indicating no substantial difference in predictive performance between the two models. In contrast, models including an interaction between meal energy and food type (M3), as well as models incorporating individual-specific random slopes for meal energy (M4), showed higher AIC values and lower support compared with M1 and M2.

These results indicate that meal energy is the primary determinant of HIF area, whereas the effect of food type on the relationship between meal energy and HIF is limited under our experimental conditions. Accordingly, we considered it appropriate to apply the more simplified model (M1) that does not explicitly account for food type when estimating energy intake from archival tag data of wild fish (Fig. 1B), which resulted in the following equation:


(8) \begin{equation*} {\displaystyle \begin{array}{c}\log \left(E\left[{\phi}_{ij}\right]\right)=4.59+0.33\;{I}_{ij}+{b}_i,\end{array}} \end{equation*}

where ${I}_{ij}$ denotes meal energy standardized to a mean of 0 and a SD of 1, and ${b}_i$ represents an individual-specific random intercept that partially accounts for among-individual variability in HIF area, including unexplained variation associated with differences in actual food consumption among individuals. The effect of meal energy on HIF area was strongly positive (β = 0.33, SE = 0.05). The intercept was estimated as 4.59 (SE = 0.11). Inter-individual variability in baseline HIF area was moderate (SD = 0.29). The dispersion parameter of the Gamma error distribution was estimated as 0.24. We used this relationship to estimate energy intake of wild yellowfin tuna from archival tag data.

### Thermal structure

**Figure 2:**
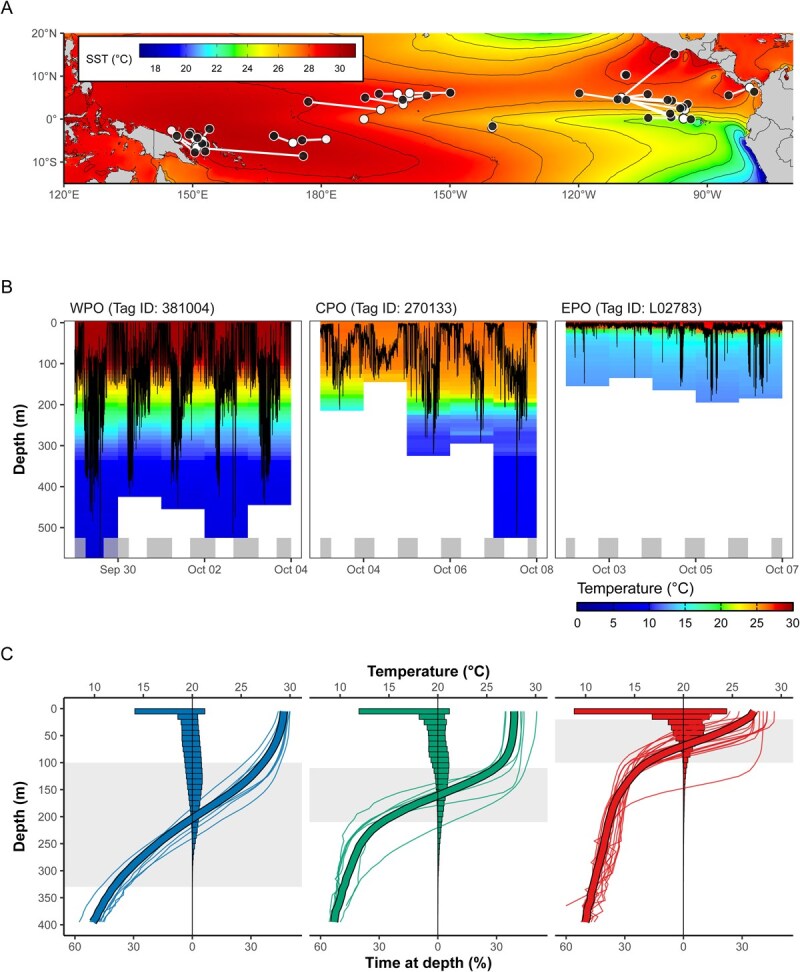
Spatial distribution, vertical behaviour and thermal environment of archival-tagged yellowfin tuna. (**A**) Release (white circles) and recapture (black circles) positions of tagged fish used in this study, shown over the mean SST field during the study period (2006–2023). (**B**) Representative examples of vertical movements (black lines) and corresponding ambient temperature profiles (colour scale) for individuals from the WPO, CPO, and EPO. Grey shaded areas represent night-time. (**C**) Temperature–depth profiles for each tagged individual within each region (thin coloured lines) together with the corresponding mean profile for each region (thick coloured line; blue = WPO, green = CPO, red = EPO). Histograms for time at depth are shown on the left (night) and right (day) for each region. Grey shaded bands denote the estimated position and thickness of thermoclines derived from the temperature–depth profiles

Across all regions, wild tagged yellowfin tuna showed a typical diel vertical pattern, remaining near the surface at night and showing repeatedly diving and surfacing behaviours during the daytime (Fig. 2B). In contrast, depth distribution differed markedly among regions. In the WPO, fish occasionally reached depths exceeding 400 m during daytime. By comparison, in the EPO, the experienced depths were largely restricted to the upper 100 m during both daytime and night-time. Daily mean ambient temperature experienced by tagged fish was broadly similar across regions despite the SST gradient, reflecting regional differences in vertical habitat use (Fig. S3).

T–D profiles derived from ambient temperature and depth records indicated regional differences in the thermal environments encountered by yellowfin tuna (Fig. 2C). Surface seawater temperatures (SSTs) were highest in the WPO (28–30°C), whereas SSTs in the EPO varied more widely, ranging from 22 to 30°C. Thermocline depth also varied among regions: mean thermocline depth was deepest in the WPO (201 m), intermediate in the CPO (164 m), and much shallower in the EPO (67.4 m), indicating the longitudinal pattern of the thermocline across the tropical Pacific Ocean.

Thermocline depth corresponded closely to the vertical thickness of the thermocline layer. In the WPO, where the thermocline occurred at greater depths, the thermocline layer extended from an upper boundary of 96.7 m to a lower boundary of 313 m, resulting in a vertical thickness of ~200 m. In contrast, in the EPO, where the thermocline was shallower, the thermocline layer spanned from 26.7 to 154 m, with a vertical thickness of ~100 m.

### Spatial distribution of energy intake

**Figure 3:**
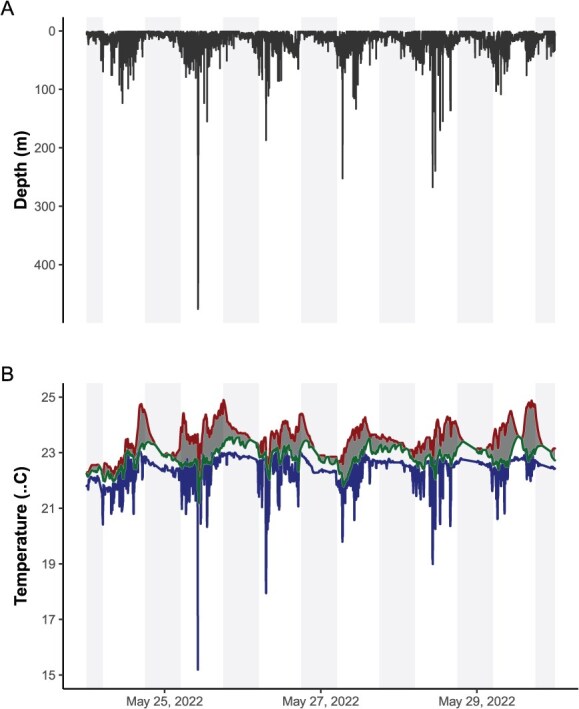
Examples of time series data of wild yellowfin tuna (Tag ID: 2190213). (**A**) Swimming depth. (**B**) Ambient temperature (blue), body temperature (red), and estimated baseline body temperature (green). Grey shaded area indicates the HIF, defined as the area where body temperature exceeds the baseline body temperature. Vertical grey bands represent night-time. HIF events were associated with vertical excursions during daytime observed in depth data

Time series trend for body temperature and estimated baseline body temperature for wild yellowfin tuna showed that body temperature occasionally increased while baseline temperature did not (Fig. 3). We considered such temperature elevations that could not be explained by ambient temperature variation as HIFs. This assumption was made based on previous studies indicating that other biological factors, such as increased swimming activity, have negligible effects on postprandial increases in visceral temperature in tunas (Clark *et al.,* 2008). We quantified these HIFs using equation (8) to estimate daily energy intake. Estimated values varied among regions, with consistently higher values towards the CPO and EPO (Fig. S5A). Regional comparison of unstandardized estimates revealed median values of 131 kcal day^−1^ in the WPO, 214 kcal day^−1^ in the CPO, and 261 kcal day^−1^ in the EPO, indicating an eastward increase in estimated energy intake (Fig. S5B).

All candidate models passed standard *sdmTMB* diagnostic checks, indicating successful convergence without numerical issues. Model selection based on AIC identified the model that included spatial terms and thermocline depth as a covariate (GM2) as the most parsimonious among the candidates (Table 2). The model that only included spatial effects (GM1) also received moderate support (ΔAIC = 1.6), whereas the model including chlorophyll a concentration (GM3) and the model without spatial effects (GM0) were less supported. While temporal and inter-annual factors were strongly confounded within region in the tagging dataset, an exploratory analysis showed that within-region variation due to season or year was relatively small (Fig. S6). As our aim was to examine the west–east spatial gradient of energy intake, these terms were therefore excluded from the geostatistical model.

**Table 2:** Model selection results for the geostatistical model fitted to energy intake (kcal day^−1^)

**Response variable**	**Model**	**Fixed effects**	**Random effects**	**AIC**	**Spatial SD (H1)**	**Spatial SD (H2)**
Energy intake	GM0	NA	id	7152.5	NA	NA
	GM1	NA	id, space	7142.5	1.16	0.41
	GM2	Thermocline depth	id, space	7140.9	0.96	0.48
	GM3	Thermocline depth, Chla	id, space	7146.8	0.98	0.40

Candidate models differ in the inclusion of spatial random effects and environmental covariates. Fixed effects include thermocline depth and chlorophyll a concentration (Chla), while random effects include individual-specific intercepts (id) and spatial random fields (space). Model performance was evaluated using AIC. Spatial SD represents the estimated SD of the spatial random field for the encounter process (H1) and the positive energy intake process (H2). NA indicates that the corresponding component was not included in the model.

In the best-fitted model (GM2), the estimated spatial SD was 0.96 for the encounter process (H1) and 0.48 for the positive energy intake process (H2). Compared with the model that only included spatial effects (GM1; Spatial SD = 1.16), the spatial SD for H1 decreased when thermocline depth was included, indicating that thermocline depth partially explained the spatial variability in the foraging probability. In contrast, spatial variability in H2 remained similar across models, indicating that substantial spatial structure in the magnitude of positive energy intake persisted even after accounting for environmental covariates. Parameter estimates from model GM2 indicated a significant negative effect of thermocline depth on the encounter process ($\alpha$ = −0.58, 95% CI = −1.08 to −0.07), with deeper thermoclines associated with a lower probability of feeding. In contrast, the effect of thermocline depth on positive energy intake overlapped zero ($\beta$ = 0.19, 95% CI = −0.04 to 0.42), indicating no significant effect on the amount of energy consumed once feeding occurred.

**Figure 4:**
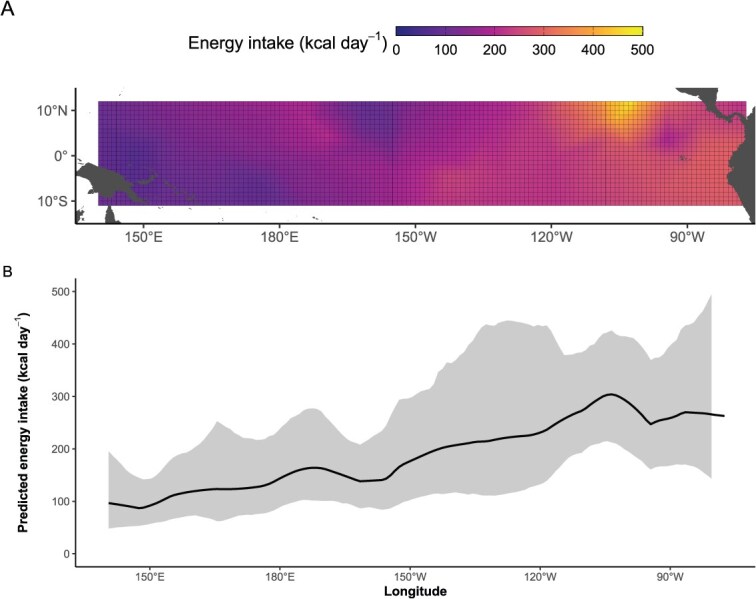
Spatial predictions of energy intake from the geostatistical model. (**A**) Spatial variation in predicted daily energy intake of yellowfin tuna across the tropical Pacific Ocean. Colours indicate the median predicted daily energy intake (kcal day^−1^) within 1° × 1° grid cells. (**B**) Longitudinal pattern of predicted energy intake derived from the model. The black line shows the mean predicted daily energy intake, and the shaded area represents the 95% confidence interval

Energy intake predicted by the best-fitted model increased from the WPO to the EPO (Fig. 4), consistent with the spatial pattern observed in the unstandardized estimates (Fig. S5A). Model-based mean energy intake among regions was estimated at 113 kcal (95% CI: 65.6–197 kcal) in the WPO, 153 kcal (95% CI: 95.0–250 kcal) in the CPO, and 245 kcal (95% CI: 151–408 kcal) in the EPO.

## Discussion

To the best of our knowledge, this study provides the first basin-scale quantification of energy intake in yellowfin tuna using archival tagging data and describes its spatial distribution across the tropical Pacific Ocean. Biologging offers a powerful means to measure physiological responses directly in free-ranging animals; however, most previous studies have focused primarily on describing behavioural patterns or environmental histories (Block et al., 1997; Schaefer and Fuller, 2022). Here, we extend the application of archival tagging data by integrating physiologically derived estimates of energy intake with a geostatistical modelling framework. This integration moves biologging data beyond individual time series towards spatially explicit physiological information, enabling inference on how energetic processes vary across oceanic regions. Our results demonstrated a clear longitudinal pattern in energy intake, with higher values estimated in the EPO. This spatial pattern suggests that regional variation in thermocline depth may be associated with differences in feeding opportunities, prey availability and energy content, and energy acquisition in yellowfin tuna. By linking biologging-derived physiological metrics to large-scale environmental structure, this study provides a physiological basis for understanding the drivers of spatial variation in growth and related demographic performance in this commercially important species.

### HIF-based approach for estimating energy intake

HIF has been used to estimate energy intake for several tuna species (Whitlock *et al.,* 2013; Aoki *et al.,* 2017; Muhling *et al.,* 2022), but has not previously been demonstrated for yellowfin tuna. Results from our feeding experiments revealed a clear relationship between ingested energy and HIF area in yellowfin tuna, demonstrating that the HIF-based approach is applicable to this species. Model selection results indicated that ingested energy was the primary determinant of HIF area, whereas differences in food type had limited effects on this relationship. This pattern is consistent with previous studies that also reported only minor effects of prey type on HIF area on the population level (Whitlock *et al.,* 2013; Aoki *et al.,* 2017). Together, these results supported the use of a simplified relationship that does not explicitly account for prey type when estimating energy intake of wild yellowfin tuna from archival tagging data.

However, interpretation of HIF-based estimates in yellowfin tuna requires careful consideration of estimation uncertainty and the spatial and temporal scales at which the method is applied. Unlike temperate tuna species, yellowfin tuna lack well-developed visceral heat exchangers (Bernal *et al.,* 2017), resulting in relatively small amplitudes of postprandial body temperature increase. Consequently, HIF signals in yellowfin tuna are more susceptible to ambient water temperature variability and measurement noise, and daily estimates of energy intake may therefore contain uncertainty. Similar patterns have been reported for skipjack tuna, which also lack visceral heat exchangers, and the relationship between ingested energy and HIF area observed in this study was more consistent with that reported for skipjack tuna (Aoki *et al.,* 2017) than for Pacific bluefin tuna (Whitlock *et al.,* 2013). For comparing HIF response among species, we used the reported regression models to estimate the expected HIF area for a standardized 100 kcal meal, which resulted in values of 335°C·min for Pacific bluefin tuna, 143°C·min for skipjack tuna, and 120°C·min for yellowfin tuna. These values indicate that the HIF response of yellowfin tuna is substantially smaller than that of bluefin tuna and comparable to that of skipjack tuna. These physiological constraints can make it more difficult to directly interpret daily HIF-based energy intake estimates in relation to fine-scale behaviour or short-term environmental variability for yellowfin tuna. In addition, the calibration derived from the feeding experiment should be interpreted as a population-level relationship because individual food consumption was estimated from cage-level feeding rather than measured directly for each fish. This can also introduce uncertainty associated with among-individual variation in actual food intake. Given these limitations, our approach is better suited to extracting large-scale spatial patterns of energy intake by averaging over individual variability and observation noise. Accordingly, this study focused on the tropical Pacific Ocean at a basin scale and applied a geostatistical framework to account for observation error and individual differences, thereby enabling robust evaluation of systematic longitudinal gradients in energy intake.

Two additional sources of uncertainty are relevant to the spatial analysis in this study. First, Whitlock *et al.* (2013) demonstrated a negative effect of ambient water temperature on HIF area in Pacific bluefin tuna, suggesting that the longitudinal gradient in ambient water temperature across the tropical Pacific Ocean could systematically bias regional estimates of energy intake. To account for this, we applied a temperature correction factor derived from Whitlock *et al.* (2013) to all HIF-based energy intake estimates prior to the geostatistical analysis. The values reported throughout this study are therefore temperature corrected. This correction resulted in a marginally larger correction in the WPO, reflecting its slightly higher ambient temperature (Fig. S3), but it did not qualitatively alter the west-to-east spatial gradient (Table S5, Fig. S4). Second, the experimental fish used for calibration (fork length: 37.6–45.7 cm, Table S2) were considerably smaller than many of the wild tagged fish (fork length: 40–135 cm, Table S3), and if the HIF–energy relationship varies with body size, absolute estimates of energy intake may be subject to some bias. However, since the primary aim of this study was to characterise relative spatial variation rather than absolute values, and since wild fish across all three regions encompassed broadly similar body size distributions (WPO: 67.6 ± 12.7 cm; CPO: 74.1 ± 17.2 cm; EPO: 63.5 ± 24.5 cm; Table S3), this uncertainty is also unlikely to affect our conclusions substantially.

### Spatial variation in energy intake and its potential drivers

Our analysis revealed a pronounced longitudinal gradient in energy intake of yellowfin tuna, with values increasing from the western to the eastern tropical Pacific Ocean. This pattern was consistently observed in both the unstandardized individual-based estimates (Fig. S5) and the predictions from the geostatistical model (Fig. 4), suggesting that it is not an artefact of the spatial distribution of tagged individuals or sampling locations. Rather, the results demonstrated a broad-scale spatial structure in energy acquisition across the tropical Pacific Ocean. Our findings suggest that longitudinal variation in oceanographic conditions may underlie spatial variation in energy intake of yellowfin tuna.

The higher energy intake estimated in the EPO appears to be related to regional differences in thermocline depth and their influence on feeding opportunities. Model selection results showed that including thermocline depth as a covariate (GM2) partially explained the spatial variability in the encounter process, although the improvement over the model that only included spatial effects (GM1) was relatively small (ΔAIC = 1.6). The negative effect of thermocline depth indicated that feeding probability increased as the thermocline depth became shallower. In such environments, key prey resources for yellowfin tuna, such as mesopelagic micronekton, are likely concentrated within a narrower depth range closer to the surface (Irigoien *et al.,* 2014). This vertical compression reduces the search space required for predators and increases encounter rates. Previous studies have reported that prey distributions in the EPO are shallower and vertically more constrained than in the western and central Pacific (Irigoien *et al.,* 2014; Aksnes *et al.,* 2017), supporting the idea that physical control of vertical habitat structure can enhance feeding efficiency. Our results are consistent with this mechanism and suggest that vertical thermal structure, particularly thermocline depth, may act as an important mediator between physical oceanography and foraging opportunity.

In contrast, including chlorophyll a concentration as a covariate did not improve model performance (GM3), indicating that surface primary productivity alone does not explain spatial variation in energy intake of yellowfin tuna. This finding is consistent with the previous study (Muhling *et al.,* 2022) that found no significant relationship between chlorophyll a and an HIF-based proxy for energy intake in North Pacific albacore tuna (*Thunnus alalunga*). Although chlorophyll a is widely used as an index of primary productivity, it does not necessarily reflect the abundance, vertical distribution, or aggregation of mesopelagic prey that constitute the main food resources for yellowfin tuna. Energy transfer from primary producers to higher trophic levels involves multiple intermediate processes, including prey behaviour, vertical migration, and physical aggregation by oceanographic features. These processes can decouple predator foraging success from surface chlorophyll patterns, particularly in pelagic systems where vertical structure plays a central role. The lack of a clear chlorophyll effect in our analysis highlights the importance of considering subsurface habitat structure rather than relying solely on surface productivity metrics.

In addition to prey density and distribution, regional differences in prey composition may also contribute to the observed spatial variation in energy intake. Previous studies investigated stomach contents of yellowfin tuna and found that fish in the WCPO consume a diverse range of prey dominated by mesopelagic fish (e.g. Myctophidae) and crustaceans, whereas diets in the EPO are less diverse but include a higher proportion of energy-rich prey such as pelagic fish (e.g. Engraulis) and cephalopods (Duffy *et al.,* 2017). Such differences in prey quality could contribute to regional variation in estimated energy intake, particularly in regions with shallow thermoclines that increase prey encounter probabilities. These findings suggest that the spatial gradient in energy intake observed in this study likely emerges from the combined effects of physical habitat structure, prey distribution, and prey quality. This integrated perspective underscores the need to link physical oceanography, trophic structure and predator physiology to understand the underlying mechanism.

### Ecological implications of spatial heterogeneity of energy intake

The observed spatial heterogeneity in energy intake has potential ecological consequences for life-history characteristics in yellowfin tuna across the tropical Pacific Ocean. Energy acquisition represents the fundamental resource that underpins survival, growth, maturation, and reproduction, and persistent regional differences in energy intake may therefore influence long-term patterns of energy allocation. Our results demonstrated that estimated energy intake was consistently higher in the EPO, suggesting that yellowfin tuna in this region may have greater energetic advantage to support somatic growth. This interpretation is consistent with reported regional differences in asymptotic fork length, with estimates of ~151 cm in WCPO (Farley *et al.,* 2020) and 188 cm in the EPO (Wild, 1986), indicating a pattern in which individuals in the eastern region attain larger body sizes. This difference in energy intake may vary ontogenetically, as fish move into the EPO region from the WCPO region or develop the physiology required to exploit prey at greater depths. Further sources of data from hard tissues, such as otoliths, may reveal chronological changes in growth across age classes (Widdrington *et al.,* 2025); while testing of these hypotheses through examination of ecosystem models could provide further evidence for the mechanistic drivers of this feeding ecology in yellowfin tuna (e.g. Senina *et al.,* 2015).

### Implications for conservation and management

Our findings have important implications for predicting the responses of tuna population dynamics to changes in the oceanographic conditions in the tropical Pacific Ocean. Several modelling studies projected that future ocean warming and the associated strengthening of upper-ocean stratification in the WCPO will reduce surface primary production and prey availability for yellowfin tuna, redistributing the population towards the EPO (Bell *et al.,* 2021; Nicol *et al.,* 2022). The relatively low energy intake estimated for WCPO yellowfin tuna in the present study suggests that foraging conditions in this region are already constrained, and such declines in prey availability may further exacerbate energetic limitations for this population. In the EPO, by contrast, the relatively higher energy intake estimated here indicated that yellowfin tuna currently experience more favourable foraging conditions. However, the recent reporting of unprecedented suppression of coastal upwelling in the EPO highlights that this productive environment can fail abruptly (O'Dea *et al.,* 2025), and more frequent occurrences of such failures in the future could reduce prey availability for yellowfin tuna even in this region. Given that foraging conditions in both regions face potential negative impacts under future climate change, monitoring spatial distribution of energy intake will be important for anticipating impacts on the yellowfin tuna population. The present spatial pattern in estimated energy intake can therefore serve as a baseline for future assessments, enabled by physiologically grounded estimation of energy intake from the HIF recorded by archival tags.

## Supplementary Material

Web_Material_coag059

## Data Availability

The data that support the findings of this study are not publicly available due to institutional restrictions but are available from the corresponding author upon reasonable request.
