# Supplementary material for: Linking physiology to seascape: spatial patterns of energy intake in a highly mobile pelagic predator inferred from electronic tags
Source: Conserv Physiol. 2026 Aug 12;14(1):coag059. doi: 10.1093/conphys/coag059 (PMC13466724; doi:10.1093/conphys/coag059)
Supplement: Supplementary file 1 [file web_material_coag059.docx]

**— Supplementary materials —**

**Linking physiology to seascape: spatial patterns of energy intake in a highly mobile pelagic predator inferred from electronic tags**

Takaaki Hasegawa, Joe Scutt Phillips, Daniel W. Fuller, Takaaki K. Abe, Kei Okamoto, Yoshinori Aoki, Kazunori Kumon, Takeshi Eba, and Takashi Kitagawa

This supplementary material provides additional tables and figures supporting the main manuscript, along with three supplementary analyses addressing potential sources of bias in the estimated spatial patterns of energy intake for yellowfin tuna. Tables S1 and S2 summarize the feeding schedule and metadata for archival-tagged fish from the feeding experiment, respectively, and Table S3 provides metadata for the archival-tagged wild fish used in the analysis. Three additional analyses are also presented: a regional residency analysis for one wild-tagged individual lacking light-based geolocation (Figure S1); the temperature correction applied to HIF-based energy intake estimates (Tables S4–S5; Figures S2–S5); and an exploration of temporal variation in energy intake, assessing whether temporal factors could account for the observed spatial pattern (Figure S6).

**Regional residency analysis for fish 340037**

One individual (fish 340037) lacked light-based geolocation for the entire track, and its release and recapture positions were very similar despite a deployment period of approximately one year. To assess whether this fish may have undertaken a cyclic migration through another region and returned, rather than remaining within the WPO throughout the deployment, we compared its thermocline depth derived from the tag against the distribution of mean thermocline depths at individual level for other tagged fish in each region.

The mean thermocline depth for fish 340037 was 196 m, within the WPO regional distribution (other individuals: 177–248 m, mean 201 ± 20.7 m, n = 9) and clearly distinct from the CPO (130–238 m, mean 164 ± 35.3 m, n = 7) and EPO (35–140 m, mean 67.4 ± 23.5 m, n = 19) distributions. Across the whole deployment period, the weekly mean thermocline depth for this fish ranged from 155 to 247 m (Figure S1), with within-individual weekly variability (SD = 17.6 m) comparable to that of other WPO individuals (median SD = 22.0 m). These results support retention of fish 340037 in the analysis as a WPO observation.

**Temperature correction applied to HIF-based estimates of energy intake**

We applied a temperature correction to all HIF-based estimates of energy intake, because the relationship between meal energy and HIF area used in this study was calibrated at a single water temperature condition (22 °C) during the feeding experiment, whereas a previous study demonstrated that HIF area in Pacific bluefin tuna decreases significantly with an increase in ambient temperature for a meal of equivalent caloric value (Whitlock et al. 2013). Because our study region was characterized by a longitudinal variation in sea surface temperature (SST, Figure 2A), we here compared daily estimates of energy intake before and after its application.

We derived a temperature correction factor from the regression slopes reported in Whitlock et al. (2013), which describe the relationship between HIF area and meal energy in Pacific bluefin tuna at three experimental temperatures (15, 20, and 22 °C) for different food types (Table S4). Because the calibration relationship used in this study was established at $T_{ref}$= 22 °C, estimated energy intake based on HIF area ($\phi$) for the original calibration is given by $\frac{\phi}{\beta\left( T_{ref} \right)}$, whereas the corresponding estimate at ambient temperature $T$ is $\frac{\phi}{\beta\left( T \right)}$. Thus, each estimate was corrected using $cf(T)= \frac{\beta\left( T_{ref} \right)}{\beta\left( T \right)}$. To obtain a single temperature-dependent correction function applicable to all observations, we calculated the mean slope across the two food types (squid and sardine) at each experimental temperature and fitted a simple linear regression to the resulting three temperature-slope pairs (Table S4), yielding $\beta\left( T \right)=0.0964 - 0.00194 \times T$. Substituting this relationship into the equation above gives the following equation:

$$\begin{aligned} cf\left( T \right)= \frac{0.0537}{0.0964 - 0.00194 \times T}, \#\left( S1 \right) \end{aligned}$$

which provides correction factor at given temperature shown in Figure S2. We applied this correction factor to each daily estimate of energy intake using the daily mean ambient temperature recorded by the archival tag.

Based on our tagging data, mean swimming depths differed among regions (Figure S3), with fish in the EPO spending more time at shallower depths compared to those in the WPO, reflecting diel vertical migration to cooler depths during the day. As a result, the mean daily ambient temperatures actually experienced by tagged fish were broadly similar across regions (Figure S3, Table S5), suggesting that vertical habitat use partially offsets the SST gradient. The WPO was approximately 1.2 °C warmer than the CPO and EPO on average, which accounts for the slightly larger temperature correction applied in that region (Table S5).

Applying the temperature correction increased energy intake estimates in all regions, with the largest relative increase observed in the WPO (+19.8%) compared to the CPO (+15.8%) and EPO (+15.2%; Table S5; Figure S4). These differences in correction magnitude reflect the slightly warmer mean temperatures recorded in the WPO. After applying the correction, the median daily energy intake increased from 109.1 to 130.7 kcal day⁻¹ in the WPO, from 184.4 to 213.5 kcal day⁻¹ in the CPO, and from 227.2 to 261.7 kcal day⁻¹ in the EPO. The ratio of median energy intake between the EPO and WPO was 2.08 before correction and remained 2.00 after correction, demonstrating that the temperature correction accounted for only a small fraction of the west-to-east spatial gradient. The regional pattern in energy intake reported throughout this study (Figure S4) was therefore not driven primarily by ambient temperature effect.

**Exploration of temporal effects on energy intake estimates**

The primary objective of this study was to characterize spatial variation in daily energy intake of wild yellowfin tuna across the tropical Pacific Ocean. Because the archival tagging data used in the analysis span from 2006 to 2023 and individual fish were tagged across different seasons and years, we explored whether temporal variation in energy intake could confound the observed spatial patterns.

Sampling coverage differed among regions in both year and season. Tagged fish in the WPO were monitored primarily between 2006 and 2013, whereas EPO fish were predominantly tracked between 2013 and 2023, with limited temporal overlap between the two regions (Table S3). CPO fish covered an intermediate period (2010-2021, Table S3). Seasonal sampling was also uneven: EPO data were concentrated in the second quarter (April-June), CPO data in the third and fourth quarters (July-October), while WPO data were distributed throughout all months (Table S3).

Despite these sampling biases, the regional pattern in daily energy intake (EPO > CPO > WPO) was consistent across all quarters and multi-year periods (Figure S6). Within each region, the interquartile ranges of energy intake overlapped substantially across quarters and periods, indicating that seasonal and interannual variation within each region was small relative to the differences among regions. These patterns suggest that the observed spatial gradient is unlikely to be an artefact of uneven temporal sampling.

We also attempted to explicitly account for temporal variation by incorporating year and quarter as explanatory variables in the geostatistical model. When year was included as a fixed effect, parameter estimates became highly unstable due to the confounding between year and region: for example, the coefficient for 2010 (the only year with data from both the CPO and EPO but not the WPO) had a standard error of 2,223, rendering the estimate unreliable. When year and quarter were instead treated as random effects, the model converged but produced a Matern range estimate of 103.8 degrees in the positive-catch component, compared with 30.1 degrees in the reference model. A range approaching the full longitudinal extent of the study area (~170 degrees) indicates that the spatial random field had effectively degenerated, with the temporal random effects absorbing much of the regional spatial signal due to the year-region confounding structure of the dataset. In both cases, the spatial inference was compromised relative to the reference model. Given these constraints, we retained the geostatistical model without explicit temporal terms as the most appropriate approach for estimating the spatial pattern in energy intake. The exploratory analysis presented above supports the conclusion that the observed regional gradient (EPO > CPO > WPO) is robust to temporal variation in observation, and is unlikely to be substantially confounded by seasonal or interannual effects.

**Tables**

**Table S1. Feeding schedule during the feeding experiment.** Daily food type and total food amount (g) provided to the sea cage are shown for each day. Daily ration (%) represents the targeted proportion of body mass relative to the total food amount supplied to the cage. Individual food consumption was estimated by dividing the total food amount by the number of tagged fish present in the cage. The table includes both the acclimatization period following tagging (13–18 February 2024) and the feeding experiment period (19–24 February 2024). In the food type column, Fish and Krill refer to sand eels (Ammodytidae) and Euphausiids, respectively

| **Date** | **Event** | **Food type** | **Food amount (g)** | **Daily ration (%)** |
| --- | --- | --- | --- | --- |
| 2024/2/13 | Tagging | Fish | 1500 | 6 |
| 2024/2/14 |  | Fish | 1500 | 6 |
| 2024/2/15 |  | Fish | 1500 | 6 |
| 2024/2/16 |  | Fish | 1500 | 6 |
| 2024/2/17 |  |  | 0 | 0 |
| 2024/2/18 |  |  | 0 | 0 |
| 2024/2/19 | Start | Fish | 1500 | 6 |
| 2024/2/20 |  | Fish | 2500 | 10 |
| 2024/2/21 |  | Fish | 500 | 2 |
| 2024/2/22 |  | Krill | 2500 | 10 |
| 2024/2/23 |  |  | 0 | 0 |
| 2024/2/24 |  | Krill | 1500 | 6 |
| 2024/2/25 |  |  | 0 | 0 |
| 2024/2/26 | End |  | 0 | 0 |

**Table S2. Summary of archival tagged yellowfin tuna for feeding experiment used in the analysis.** For each individual, fork length (FL) and body weight (BW) at the end of the experiment are shown

| **Tag ID** | **FL (cm)** | **BW (kg)** |
| --- | --- | --- |
| 2672 | 44.1 | 1.48 |
| 2850 | 39.3 | 1.00 |
| 2981 | 39.8 | 1.12 |
| 4389 | 41.3 | 1.20 |
| 4393 | 44.1 | 1.64 |
| 4394 | 42.2 | 1.32 |
| 4395 | 40.9 | 1.26 |
| 4396 | 39.4 | 1.00 |
| 4452 | 44.8 | 1.60 |
| 4453 | 45.7 | 1.80 |
| 4454 | 39.6 | 1.20 |
| 4459 | 41.4 | 1.24 |
| 4460 | 38.0 | 0.94 |
| 4461 | 39.6 | 0.98 |
| 4462 | 41.0 | 1.22 |
| 4463 | 40.5 | 1.16 |
| 4465 | 37.6 | 0.88 |
| 4976 | 40.3 | 1.14 |
| 4977 | 38.1 | 0.86 |
| 4978 | 38.7 | 1.04 |

## Table S3. Summary of archival tagged wild yellowfin tuna used in this study. For each individual, release and recapture information, days at liberty, and the geolocation method used to estimate daily positions are shown

|  | | | **Release** | | | | **Recapture** | | | |  |  |
| --- | --- | --- | --- | --- | --- | --- | --- | --- | --- | --- | --- | --- |
| **Source** | **Region** | **Fish ID** | **Date** | **Lon.** | **Lat.** | **FL** | **Date** | **Lon.** | **Lat.** | **FL** | **Days at  liberty** | **Method** |
| SPC | WPO | 9846 | Sep 24 2006 | 146.44°E | 4.34°S | 98 | Jan 26 2007 | 150.53°E | 7.77°S | 117 | 124 | TrackIt |
| SPC | WPO | 281014 | Apr 25 2011 | 150.65°E | 6.67°S | 51 | Mar 10 2012 | 146.32°E | 3.91°S | 94 | 320 | TrackIt |
| SPC | WPO | 382162 | Apr 20 2013 | 150.79°E | 6.53°S | 72 | Aug 30 2013 | 149.18°E | 3.66°S | 85 | 132 | TrackIt |
| SPC | WPO | 340038 | Jan 29 2012 | 150.81°E | 6.28°S | 63 | Mar 27 2012 | 153.93°E | 2.29°S | 64 | 58 | TrackIt |
| SPC | WPO | 340037 | Jan 29 2012 | 150.81°E | 6.28°S | 66 | Jan 17 2013 | 152.24°E | 5.71°S | 115 | 354 | NA |
| SPC | WPO | 340034 | Jan 29 2012 | 150.81°E | 6.28°S | 64 | Dec 16 2012 | 149.51°E | 3.17°S | 94 | 322 | ukfsst |
| SPC | WPO | 340030 | Jan 29 2012 | 150.81°E | 6.28°S | 64 | Dec 24 2012 | 151.13°E | 4.40°S | 71 | 330 | TrackIt |
| SPC | WPO | 381004 | Apr 19 2013 | 150.83°E | 6.52°S | 72 | Nov 1 2013 | 152.95°E | 7.53°S | 93 | 196 | TrackIt |
| SPC | WPO | 382297 | Apr 20 2013 | 150.93°E | 6.69°S | 71 | Dec 22 2013 | 175.81°E | 8.60°S | 106 | 246 | TrackIt |
| SPC | WPO | 339748 | Jan 27 2012 | 152.39°E | 4.98°S | 55 | Nov 17 2013 | 152.95°E | 6.02°S | 94 | 660 | ukfsst |
| SPC | CPO | 476905 | Jul 21 2021 | 159.43°W | 6.07°N | 54 | Aug 8 2021 | 149.91°W | 6.13°N | 56 | 18 | NA |
| SPC | CPO | 478925 | Jul 28 2021 | 160.88°W | 3.44°N | 75 | Sep 19 2021 | 169.77°W | 4.98°N | 75 | 53 | NA |
| SPC | CPO | 270133 | Jun 5 2010 | 162.18°W | 5.88°N | 79 | Apr 3 2011 | 161.00°W | 4.50°N | 98 | 302 | WLC |
| SPC | CPO | 270134 | Jun 5 2010 | 162.18°W | 5.88°N | 98 | Oct 6 2011 | 166.55°W | 5.88°N | 119 | 488 | TrackIt |
| SPC | CPO | 481510 | Aug 4 2021 | 166.11°W | 2.19°N | 57 | Sep 7 2021 | 176.95°E | 4.03°N | 61 | 34 | NA |
| SPC | CPO | 320349 | Oct 14 2011 | 170.04°W | 0.03°S | 63 | Nov 6 2011 |  |  | 63 | 23 | NA |
| SPC | CPO | 450248 | Aug 14 2018 | 178.83°W | 4.68°S | 93 | Dec 1 2018 | 175.53°E | 4.94°S | 115 | 109 | ukfsst |
| IATTC | EPO | D4387 | Sep 15 2009 | 80.13°W | 7.32°N | 67 | May 27 2010 | 79.07°W | 6.37°N | 92 | 254 | ukfsst |
| IATTC | EPO | D0764 | Jan 30 2007 | 80.13°W | 7.32°N | 58 | Apr 21 2009 | 85.03°W | 5.53°N | 127 | 812 | ukfsst |
| IATTC | EPO | L02785 | Apr 11 2022 | 95.53°W | 0.09°N | 47 | Jul 11 2022 | 93.83°W | 0.05°S | 53 | 91 | ukfsst |
| IATTC | EPO | 2190192 | Apr 11 2022 | 95.53°W | 0.09°N | 69 | Jun 5 2022 | 95.72°W | 2.70°N | 72.5 | 55 | ukfsst |
| IATTC | EPO | L02795 | Apr 11 2022 | 95.53°W | 0.09°N | 47 | Jul 15 2022 | 108.88°W | 4.47°N | 58 | 95 | ukfsst |
| IATTC | EPO | 2190190 | Apr 16 2022 | 95.53°W | 0.11°N | 60 | May 27 2022 | 98.67°W | 1.27°N | 63 | 41 | ukfsst |
| IATTC | EPO | 2190213 | Apr 5 2022 | 95.55°W | 0.07°N | 74 | Jul 6 2022 | 96.32°W | 2.50°N | 78.5 | 92 | ukfsst |
| IATTC | EPO | 1890108 | Apr 15 2019 | 95.60°W | 0.18°N | 64 | Jun 20 2019 | 103.78°W | 0.27°N | 73 | 66 | ukfsst |
| IATTC | EPO | B1180 | Mar 7 2013 | 109.17°W | 10.32°N | 135 | Sep 17 2013 | 109.17°W | 10.32°N | 172 | 194 | ukfsst |
| IATTC | EPO | B1156 | Mar 5 2013 | 109.17°W | 10.32°N | 104 | Sep 17 2013 | 109.17°W | 10.32°N | 125 | 196 | ukfsst |
| IATTC | EPO | B1204 | Mar 4 2013 | 109.17°W | 10.32°N | 88 | Sep 20 2013 | 108.93°W | 10.28°N | 100 | 200 | ukfsst |
| IATTC | EPO | L02783 | May 3 2022 | 110.05°W | 5.00°N | 42 | Apr 20 2023 | 97.65°W | 15.10°N | 92.5 | 352 | ukfsst |
| IATTC | EPO | L02793 | May 3 2022 | 110.05°W | 5.00°N | 45 | Sep 21 2022 | 98.42°W | 0.40°N | 57.1 | 141 | ukfsst |
| IATTC | EPO | L02659 | May 3 2022 | 110.05°W | 5.00°N | 46 | Aug 6 2022 | 98.25°W | 4.28°N | 52 | 95 | ukfsst |
| IATTC | EPO | L02786 | May 3 2022 | 110.05°W | 5.00°N | 40 | Jul 29 2022 | 99.25°W | 4.47°N | 48.5 | 87 | ukfsst |
| IATTC | EPO | L02628 | May 3 2022 | 110.05°W | 5.00°N | 45 | Jun 4 2022 | 110.88°W | 4.60°N | 48.5 | 32 | ukfsst |
| IATTC | EPO | L02719 | May 3 2022 | 110.06°W | 5.00°N | 48 | Sep 17 2022 | 94.58°W | 3.53°N | 55.8 | 137 | ukfsst |
| IATTC | EPO | L01441 | Apr 28 2019 | 110.07°W | 4.97°N | 48 | Aug 19 2019 | 103.90°W | 5.80°N | 59 | 113 | ukfsst |
| IATTC | EPO | A0121 | Oct 24 2009 | 140.17°W | 2.03°S | 79 | Feb 2 2010 | 140.08°W | 1.67°S | 94 | 101 | ukfsst |

**Table S4. Regression slopes estimated by different ambient temperature levels reported by Whitlock et al. (2013)**. $\beta$ represent slope parameters estimated for Pacific bluefin tuna by different food types.

| **Temperature (°C)** | $\boldsymbol{\beta}$ **(Squid)** | $\boldsymbol{\beta}$ **(Sardine)** | $\boldsymbol{\beta}$ **(Mean)** |
| --- | --- | --- | --- |
| 15 | 0.075 | 0.057 | 0.066 |
| 20 | 0.071 | 0.053 | 0.062 |
| 22 | 0.056 | 0.045 | 0.051 |

**Table S5. Summary of ambient temperature conditions and the effect of temperature correction on daily energy intake estimates of wild yellowfin tuna by region.** Ambient temperature is the mean daily ambient temperature recorded by archival tags. Correction factor was calculated using the equation S1. Energy intake and corrected energy intake are regional median daily values before and after correction, respectively. Change is the difference in median energy intake following correction shown in percentage.

| **Region** | **Ambient temperature  (°C)** | **Correction factor** | **Energy intake (kcal day⁻¹)** | **Corrected energy intake (kcal day⁻¹)** | **Change (%)** |
| --- | --- | --- | --- | --- | --- |
| WPO | 26.7 ± 1.4 | 1.21 | 109.1 | 130.7 | +19.8 |
| CPO | 25.6 ± 1.7 | 1.16 | 184.4 | 213.5 | +15.8 |
| EPO | 25.5 ± 1.9 | 1.15 | 227.2 | 261.7 | +15.2 |

**Figures**

**Figure S1.** Weekly mean thermocline depth for fish 340037 relative to the regional distributions (mean ± SD across other individuals) for WPO, CPO, and EPO.

**Figure S2. Temperature correction factor as a function of ambient temperature.** The solid line shows the fitted relationship from Whitlock et al. (2013). Dashed line: factor = 1; dotted line: reference temperature ($T_{ref}$) calibrated from the feeding experiment (22 °C).

**Figure S3. Regional comparisons of daily mean ambient temperature (left panel) and mean swimming depths (right panel) by daytime** **(Day), nighttime (Night), and daily mean (Daily mean)****.** Boxes show the interquartile range (IQR), horizontal lines indicate medians, whiskers extend to 1.5 × IQR, and outliers are plotted individually.

**Figure S4. Daily energy intake estimates by region before (Original) and after (Corrected) temperature correction.** Boxes show the interquartile range (IQR), horizontal lines indicate medians, whiskers extend to 1.5 × IQR, and outliers are plotted individually.

**Figure S5. Spatial comparison of HIF-based energy intake.** (A) Spatial variation in daily energy intake of wild yellowfin tuna in the tropical Pacific Ocean after the temperature correction. Colors represent the median energy intake (kcal day⁻¹) within 1° × 1° grid cells. (B) Regional comparison of daily energy intake estimated from the heat increment of feeding (HIF). Median energy intake (kcal day⁻¹) is shown for the Western Pacific Ocean (WPO, N = 10), Central Pacific Ocean (CPO, N = 7), and Eastern Pacific Ocean (EPO, N = 19) across individuals. Horizontal lines indicate the median, boxes represent the interquartile range (25th–75th percentiles), and whiskers extend to the 2.5th and 97.5th percentiles

**Figure S6. Daily energy intake estimates for wild yellowfin tuna by region**, grouped by quarter (left panel, Q1: January-March, Q2: April-June, Q3: July-September, Q4: October-December) and four-year period (right panel). Only days with positive energy intake are shown. Boxes show the interquartile range (IQR), horizontal lines indicate medians, whiskers extend to 1.5 x IQR, and outliers are plotted individually.
